# Field resistance of transgenic plantain to nematodes has potential for future African food security

**DOI:** 10.1038/srep08127

**Published:** 2015-01-30

**Authors:** Leena Tripathi, Annet Babirye, Hugh Roderick, Jaindra N. Tripathi, Charles Changa, Peter E. Urwin, Wilberforce K. Tushemereirwe, Danny Coyne, Howard J. Atkinson

**Affiliations:** 1 International Institute of Tropical Agriculture, Nairobi, Kenya; 2 International Institute of Tropical Agriculture, Kampala, Uganda; 3 Centre for Plant Sciences, University of Leeds, Leeds LS2 9JT, UK; 4 National Agriculture Research Laboratories, Kampala, Uganda

## Abstract

Plant parasitic nematodes impose losses of up to 70% on plantains and cooking bananas in Africa. Application of nematicides is inappropriate and resistant cultivars are unavailable. Where grown, demand for plantain is more than for other staple crops. Confined field testing demonstrated that transgenic expression of a biosafe, anti-feedant cysteine proteinase inhibitor and an anti-root invasion, non-lethal synthetic peptide confers resistance to plantain against the key nematode pests *Radopholus similis* and *Helicotylenchus multicinctus*. The best peptide transgenic line showed improved agronomic performance relative to non-transgenic controls and provided about 99% nematode resistance at harvest of the mother crop. Its yield was about 186% in comparison with the nematode challenged control non-transgenic plants based on larger bunches and diminished plant toppling in storms, due to less root damage. This is strong evidence for utilizing this resistance to support the future food security of 70 million, mainly poor Africans that depend upon plantain as a staple food.

Banana and plantain (*Musa* spp.) are cultivated in over 130 countries worldwide covering approximately 10 million hectares, with an annual production of 139 million tons^1^. They rank 8^th^ in world production of staple food crops^2^. Often they are produced by small-scale farmers in tropical and subtropical regions mainly for home consumption and also for sale in local and regional markets rather than for international trade. For instance, in South-eastern Nigeria, smallholder farmers generate up to 30% of their income from plantain (*Musa* spp. AAB genome) cultivation^3^. In Central and West Africa, plantains account for about 32% of total *Musa* production^4^, which feed approximately 70 million people with >25% of their carbohydrates and 10% of their food energy^5^,^6^. Closing the yield gap of staple crops is a priority for ensuring future world food security^7^. A recent analysis suggests West Africa and in particular Southern Nigeria is a key area where that is needed^8^. Banana and plantain feed more people there per unit area than other staple crops and are cheaper to produce than rice or wheat^9^.

Black sigatoka and nematodes are the major biotic constraints affecting plantain production in West and Central Africa^10^,^11^. Black sigatoka (caused by *Mycosphaerella fijiensis*) accounts for losses of about 40%, while nematodes are responsible for losses of about 31–50% with current yields of 7.8 metric tonnes per hectare^12^. Losses due to nematodes are most severe when storms cause toppling to plants that have root systems damaged by them. Weevils (*Cosmopolites sordidus)* are also considered an important pest of bananas and plantains with reported yield losses of up to 40%^13^,^14^. *Banana Bunchy Top Virus* (BBTV) is also emerging as one of the important pathogens of plantain in West and Central Africa^15^.

Nematodes are often controlled in commercial plantations by periodic application of pesticides which are environmentally unacceptable. Analysis of data from experimental applications of nematicides across a range of African countries has demonstrated the considerable impact of nematodes on *Musa* across the continent^16^, such as yield responses of 71 ± 16% over three years after nematicide application^17^. However, access to suitable, high quality nematicides is often not reliable for smallholder producers in Africa, while many of the products traditionally used have since been withdrawn from use due to their high toxicity and environmental unacceptability^18^,^19^. A second approach of crop rotation is not often possible for farmers with insufficient land to accept the associated yield loss, because plantains out-produce all other staple crops in conditions that favour them.

The key nematode pests of *Musa* spp. are *Radopholus similis*, *Pratylenchus goodeyi*, *P. coffeae*, *Helicotylenchus multicinctus* and *Meloidogyne* spp. *R. similis* is a migratory endoparasite causing root necrosis which pre-disposes the plant to topple during storms and considered the most damaging species, although it often occurs in mixed populations with other species^16^. Furthermore, *Pratylenchus* spp. are becoming increasingly prevalent pests of *Musa* across Africa, especially on plantain in West Africa, resulting in growing concern for their potential impact^20^. *P. coffeae* and *P. goodeyi* impose root pathology similar to *R. similis* and are major pests wherever they occur^21^. *H. multicinctus* occurs very widely and also feeds destructively on roots causing some root necrosis, unlike the sedentary parasite *Meloidogyne* spp. that modifies plant cells into a feeding site at one locale^22^. The combination of nematode species present varies with locality with mixtures of *P. coffeae*, *H. multicinctus*, *R. similis* and *Meloidogyne* spp. often occurring in West African soils^23^.

Cultivated plantains are sterile with triploid genomes^24^ that hamper improvement by traditional cross-pollination techniques. Although conventional breeding has produced hybrids with resistance against nematodes^25^, this is only effective against single species and not against concurrent infections by different nematodes due to the limited genetic base in *Musa* for nematode resistance^24^,^26^,^27^. Broad spectrum resistance is required to facilitate management of multiple nematode species challenging the plantain crop. Transgenic approaches have considerable potential for sustainable improvement of plantain. Due to a lack of cross-fertile wild relatives in many plantain-producing areas, as well as the male and female sterility of most edible cultivars and clonal mode of propagation, gene flow is not an issue for this crop, making a transgenic approach even more attractive.

One approach to transgenic resistance to nematodes involves disrupting their feeding. Cysteine proteinases are major digestive enzymes of many nematodes and small protein inhibitors (cystatins) from plants have mediated nematode resistance when expressed in several crops including tomato^28^, *Arabidopsis*^29^,^30^, rice^31^, potato in the field^32^,^33^,^34^, banana^35^ and plantain^36^. A second form of transgenic resistance reduces nematode invasion and its concomitant damage in addition to reducing the population that develops in roots. Two non-lethal synthetic peptides are known to have this effect^37^,^38^. The transgenic plants secrete peptides from their roots due to an N-terminal cleaved extracellular export signal^37^. Uptake of these peptides by the nematodes and suppression of invasion, therefore, presumably occurs in soil at or near the rhizoplane prior to invasion but may also impair orientation within the root. Transgenic potato plants that secreted one peptide from their roots under control of the constitutive CaMV35S promoter reduced the establishment of the potato cyst nematode *Globodera pallida*^37^. The same peptide provided 94.9 ± 0.8% resistance to *G. pallida* in a glasshouse trial when expressed in transgenic potato plants under control of a root-cap-specific promoter^39^. The second peptide, used in the current work, is a disulphide-constrained 7-mer with the amino sequence CTTMHPRLC^36^,^38^. It provided resistance to *G. pallida* in the glasshouse and field under regulation of a root-cap-specific promoter^40^. Fluorescent tagging of this peptide showed that it is taken up via chemosensory sensillae in the amphidial pouches of cyst nematodes^41^. It undergoes retrograde transport along sensory dendrites to neuronal cell bodies resulting in a loss of orientation to roots. A similar uptake pathway was also observed for *R. similis* and the peptide conferred resistance in plantain to this nematode and *H. multicinctus* in screen house trials^36^. The peptide is not persistent in soil^36^ and the biosafety of both transgenes has been studied in detail without adverse effects being detected^42^.

About 250 independent transgenic lines of the plantain cultivar ‘Gonja manjaya' were generated using a maize cystatin and synthetic peptide either singly or by stacking these genes^36^. These lines were characterized at the molecular level and evaluated in the screen house for resistance against nematodes. The study showed that the maize cystatin and synthetic peptide are capable of providing resistance in plantain to concomitant infection with different nematode species in the screen house conditions. Twelve promising plantain transgenic lines, expressing the cystatin, peptide or both genes, were selected for further field trial evaluation based on molecular analysis and subsequent screen house trials. Here, we report that several of these lines successfully provided nematode resistance, reducing nematode damage and resulting in better plant growth and yield under field conditions. This provides the first field-based demonstration of transgenic resistance against nematodes in *Musa* spp. and offers a basis for effective control of nematodes on plantains in Africa.

## Results

Twelve independent lines [5 lines with synthetic peptide (P lines), 2 lines with maize cystatin (C lines) and 5 lines with stacked dual genes (D lines)] of plantain cultivar ‘Gonja manjaya' (AAB), were evaluated in a confined field trial at the National Agriculture Research Laboratories (NARL), in Uganda following approval from the National Biosafety Committee (NBC). They were selected from the large number of transgenic lines originally generated on the basis of screen house evaluations and detectable expression of the transgene(s). The data presented here is for transgenic plants that had nematodes added to their pot soil one month before transplanting to the field. The results also include a similarly challenged set of non-transgenic control plants and second set not receiving nematodes before planting.

### Nematode densities on transgenic and non-transgenic control plants

**Figure 1:**
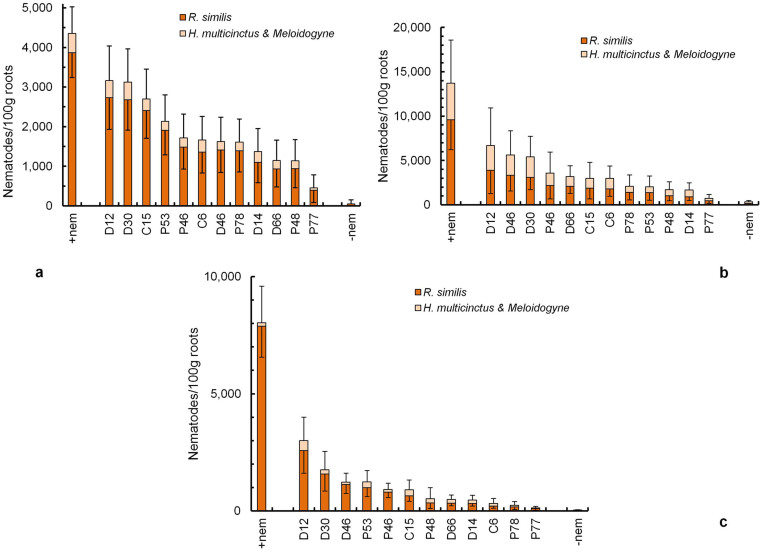
Nematode densities recovered from roots of plantain transgenic lines and control non-transgenic plants to which the nematodes were added (+nem) or not added (−nem) before planting. The bar charts are (a) during vegetative growth of the plant; (b) at flowering and (c) at harvest. The values are means ± SEM.

Nematode densities in roots of transgenic lines and control non-transgenic plants were assessed at five time points [4, 7, 10 months after planting (MAP), at flowering and harvest]. Flowering and harvest dates varied slightly for each line. Univariate analysis established that there were significant differences among lines and also blocks in nematode densities but there was no significant interaction of these two factors. Differences in block means were probably due to the negative effect on the plantains of a termite colony that was active close to the trial site adjacent to one block. The analysis established that nematode density per line did not change significantly during vegetative growth; therefore, the data collected at 4, 7 and 10 MAP were combined for further analysis. The control plants with added nematodes had a mean density of 4,351 ± 678 nematodes/100 g roots during vegetative growth. This value increased significantly by flowering to 13,712 ± 4,868 nematodes/100 g roots and was 7,883 ± 1,565 nematodes/100 g roots at harvest. However, there were significantly fewer nematodes in roots of all transgenic lines by harvest (Fig. 1).

**Figure 2:**
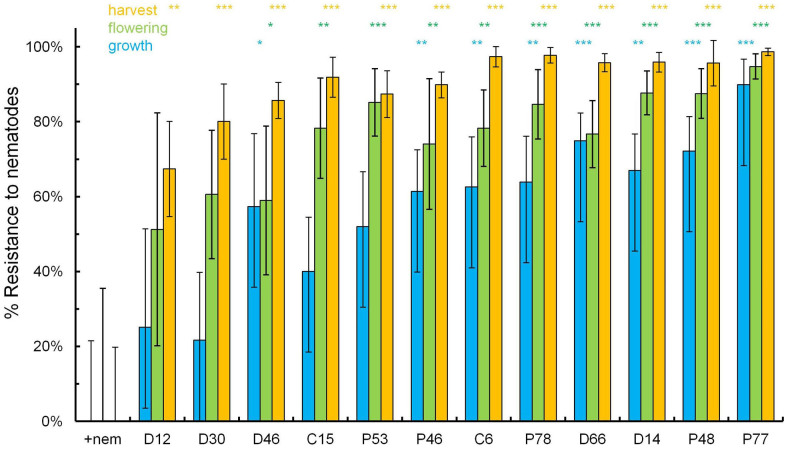
Estimated mean level of resistance (±SEM) over the period of vegetative growth, flowering and harvest (left to right) for each transgenic line relative to the control plants to which nematodes were added before planting (+nem). Each line is compared for the three time points to the corresponding control using oneway ANOVA with *apriori* contrasts (***, P < 0.001; **, P < 0.01 and *, P < 0.05).

Resistance levels of the transgenic lines are expressed on a percentage basis from the proportion of [1- (density of nematodes on a transgenic line/mean density for nematode on the challenged control plants)]. The percentage resistance was statistically significant for eight lines (C6, D14, D46, D66, P46, P48, P77 and P78) at all three assessments made during vegetative growth, at flowering and at harvest (Fig. 2). The majority of the nematodes associated with the roots were *R. similis*. The numbers of *H. multicinctus* recovered from the roots of the control plants with added nematodes were 174 ± 74, 3,865 ± 1,492 and 478 ± 178 nematode/100 g roots during vegetative growth and at flowering and at harvest, respectively. The corresponding means for the most resistant line P77 were only 5.82 ± 4.6, 230 ± 174 and 2.4 ± 4.4 *H. multicinctus*/100 g roots, which were not significantly different from the numbers of *H. multicinctus* recovered from control plants without added nematodes. Even fewer *Meloidogyne* spp. were recovered. The highest number of motile nematodes of *Meloidogyne* spp. on the control with added nematodes was only 3.53/100 g roots during vegetative growth and was usually lower at flowering and harvesting time for all the transgenic lines. These means were too low for detailed statistical analysis.

### Root necrosis and nematode densities

**Figure 3:**
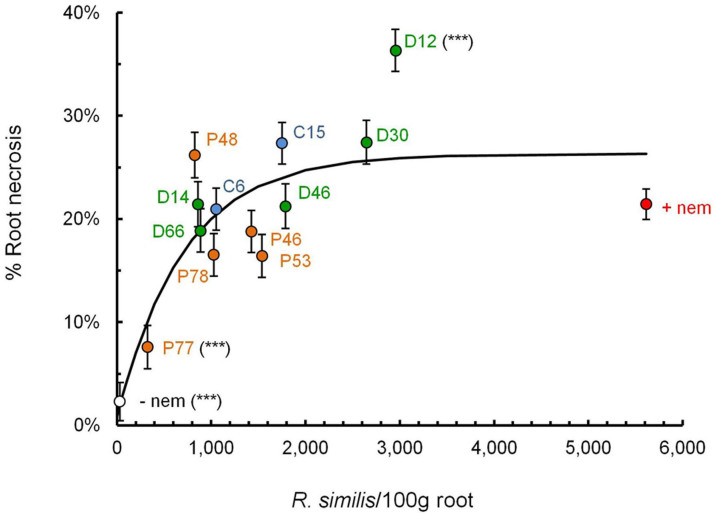
The relationship between estimated root necrosis (%) and *R. similis* densities for transgenic lines and non-transgenic control plants in the confined field trial. The slope is determined using asymptotic regression. Transgenic lines that differ significantly from the control plants with added nematodes (+nem) using Bonferroni test (modified LSD) for multiple comparisons in univariate ANOVA are shown (***, P < 0.001). The control to which nematodes were not added (−nem) before planting is provided for comparison.

The root necrosis for all the transgenic lines was compared to the control plants with added nematodes. It was assessed at the five time points listed above and Univariate ANOVA was performed to provide means adjusted for the covariate MAP. Only two transgenic lines showed significantly different root necrosis from that of the control plants with added nematodes (Bonferroni test for multiple comparisons, Fig. 3). Line P77 significantly suppressed necrosis to 7.6 ± 2.1%, whereas roots of D12 were 36.3 ± 2.0% necrotic and significantly more than the 21.4 ± 1.5% for the control plants with added nematodes. An asymptotic curve fitted by non-linear regression provided a significant fit to the data (R^2^ = 0.663, n = 14, df = 12, P < 0.001; Fig. 3).

### Nematode densities, leaf area index and yield

**Figure 4:**
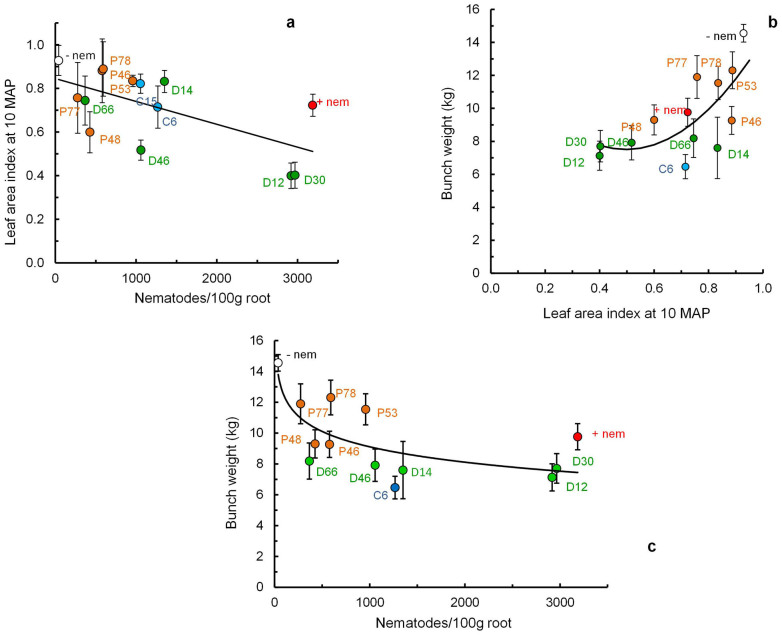
(a) Relationship between nematode density and leaf area index (LAI) at the end of vegetative growth (10 MAP) and the data fits a linear regression; (b) Relationship between leaf area index at 10 MAP and harvested bunch weight about 5 months later and a quadratic curve is fitted; (c) The relation between nematode density at the end of vegetative growth (10 MAP) and yield of the harvested bunch weight about 5 months later and the data fits a logarithmic regression.

Leaf area index (LAI) is widely used to measure the growth of plants and can be defined as the single-sided green leaf surface area per-unit area of the ground. It relates to the capacity of the banana canopy to intercept solar radiation and fix carbon^43^. We have previously measured LAI of plantains by hemispherical digital photography^22^. Regression analysis established that the LAI at the end of the vegetative growth phase (10 MAP) declined significantly in a linear manner with increasing nematode density (Fig. 4a; P < 0.014, n = 14, df = 12, for significance of the slope). The increase in bunch weight at harvest significantly correlated with the increase in LAI at the end of the vegetative growth phase with a quadratic curve rather than linear relationship providing the best fit (Fig. 4b; P = 0.038, n 13, df = 11). Correspondingly, increased nematode density significantly correlated with decreased bunch weight with a logarithmic curve (Fig. 4c; P = 0.005, n = 13, df 11).

### Nematode densities, agronomic and yield performance

Frequent observation did not reveal differences in morphology between transgenic and non-transgenic control plants during the field trial. Oneway ANOVA with *apriori* contrasts established that the control plants without added nematodes outperformed the control plants with added nematodes in six of the nine agronomic parameters with mean values assessed in Table 1. This indicates that inoculating nematodes before planting ensured a subsequent, considerable biotic stress on the plants in the field. Overall, the transgenic lines did not perform as well as the control plants without added nematodes, however, some lines significantly outperformed the control plants with added nematodes for the mean number of functional leaves (D14, P78), leaf area (D14, D66, and P48), bunch weight (P78) and number of hands (P77) (Table 1). A few of the transgenic lines did not perform as well as the control plants with added nematodes for most of the factors. The most extreme cases were line D12 with six parameters measuring significantly less than the control with added nematodes and C15 with three such parameters. The yielding plants column in Table 1 provides the number of plants that did not topple of those planted and so yielded a bunch at harvest. Both P53 and P77 outperformed the control plants with added nematodes (χ^2^; P < 0.05 and P < 0.01 respectively) whereas the toppling incidence was greater for D30 and D12 (χ^2^; P < 0.05 and P < 0.01) than for the added nematode controls.

**Figure 5:**
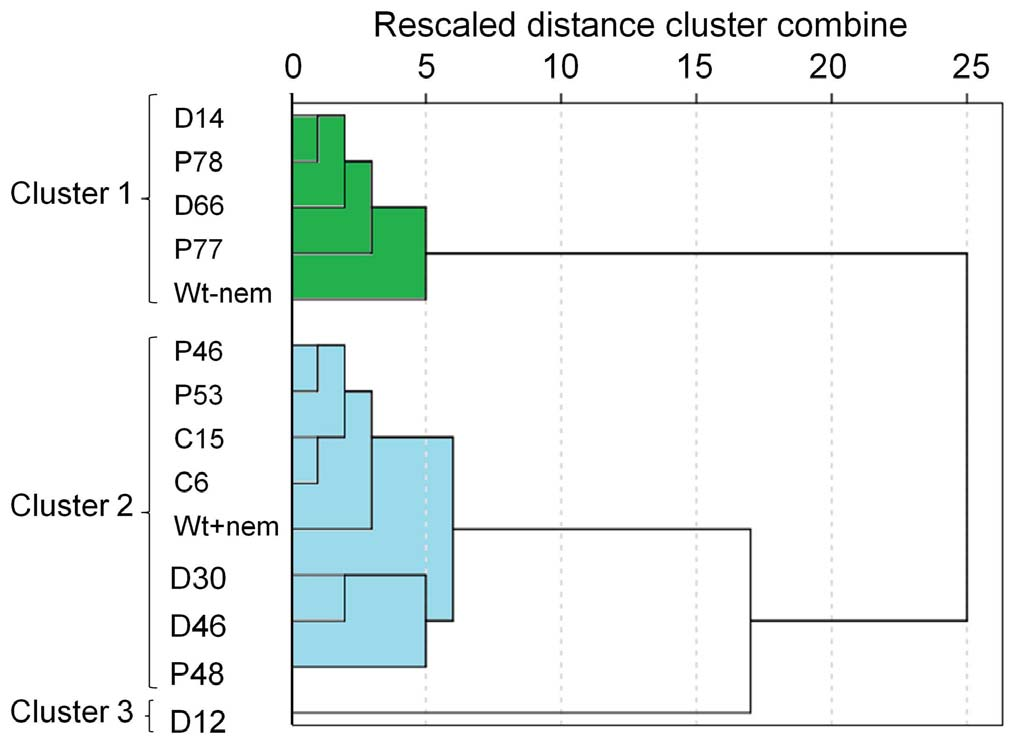
A dendrogram from cluster analysis based on the means provided in Table 1 and Fig. 2.

Cluster analysis was conducted to reveal associations and structure for all the data, and to identify transgenic lines of interest for further study (Table 1 and Fig. 2). A Hierarchical approach clustered four transgenic lines (D14, D66, P77 and P78) with high resistance against nematodes in Fig. 2 in a cluster with the control plants without added nematodes (Fig. 5). This suggests their levels of resistance were sufficient to lessen the impact of nematodes on these plants. In contrast, all other lines (C6, C15, D30, D46, P46, P48 and P53) except D12 clustered with the control plants with added nematodes indicating that their level of nematode resistance and plant agronomic parameters was insufficient to group them separately from these control plants. Line D12 was clustered alone as very distinct from other lines (Fig. 5). It provided the lowest level of resistance and showed a poor agronomic performance (Table 1 and Fig. 2).

### Molecular Characterization

**Figure 6:**
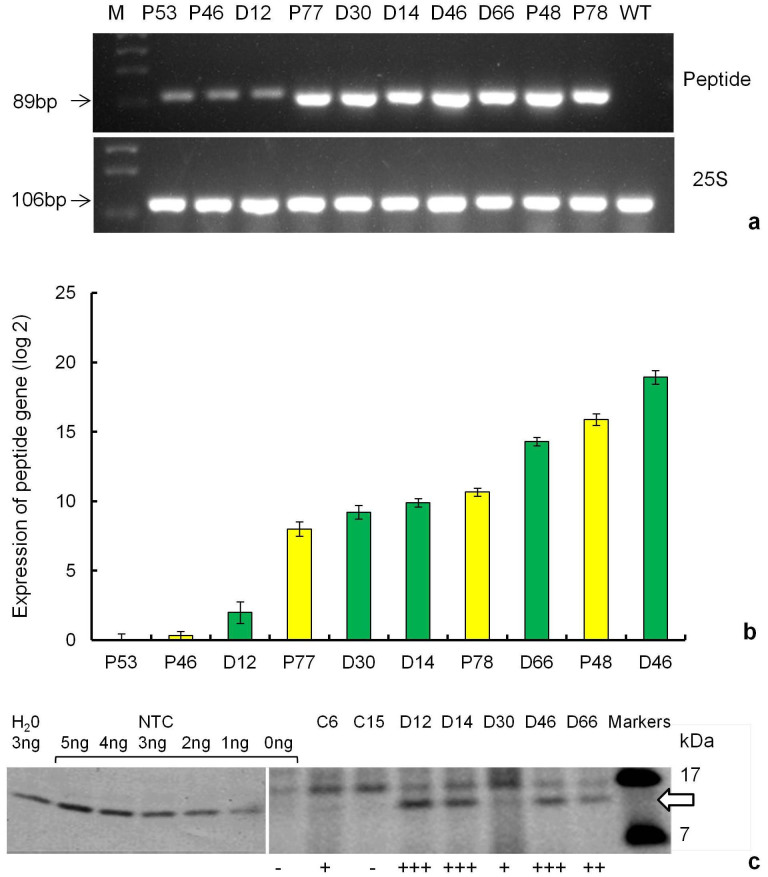
Molecular analysis of transgenic lines under evaluation in confined field trial. (a) RT-PCR of 10 transgenic plantain lines (peptide only and dual stacked genes lines) using primers specific to peptide and *25S* ribosomal gene. The figure shows cropped gels and both gels were run under the same experimental conditions. Amplified RT-PCR product designations were shown on the right and products sizes were shown on the left. M - molecular weight marker, NTC - non-transgenic control; (b) Relative transcript levels of the peptide in 10 transgenic lines (peptide only and dual stacked genes lines). Expression of peptide was normalized with banana *25S* ribosomal gene (internal control) and the non-transgenic plant served as a calibrator. Relative expression was determined from replicate measurements in two independent biological replicates and three technical replicates. Values are means ± SEM. (c) Detection of cystatin expression by western blot for seven transgenic plantain lines (cystatin lines and dual stacked genes line) using protein extracted from roots pooled of four replicate plants in each block of the contained field trial. The molecular weight of the transgenic maize cystatin is indicated (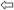). Non-transformed Gonja manjaya control (NTC) protein extract was spiked with the indicated quantity of 6 × His-tagged cystatin for positive controls. The figure shows cropped blots and gels were run under the same experimental conditions.

Total RNA was extracted from all the dual transgenic lines and peptide lines and analysed for expression levels of the synthetic peptide by RT-PCR and qRT-PCR using gene-specific primers. The amplified peptide transcript was observed in all the transgenic lines tested confirming expression of transgene in all the tested lines (Fig. 6a). The *25S* ribosomal transcripts amplification of the internal control was also detected in all the plants tested. Quantitative RT-PCR, established variation in the peptide transcript level (Fig. 6b); however, there was no overall a correlation between transcript level and nematode resistance. No transcripts corresponding to the transgene were found in the RNA isolated from non-transgenic control plants.

Proteins extracted from cystatin lines (C6 and C15) and dual stacked genes lines (D12, D14, D30, D46 and D66) were analysed by western blot to confirm the expression of the cystatin gene (Fig. 6c). The expression of cystatin was above detectable levels for all these lines except C15. Expression levels in the dual cystatin lines were equivalent to 0.013–0.033% of total plant protein loaded onto the gel.

## Discussion

Genetic engineering can add desirable traits to banana and plantain and circumvent the long crossing cycles that prevail for their traditional breeding programs. For example, Tripathi *et al.*^44^ demonstrated development of genetically modified banana with field-based evidence for resistance to the bacterial pathogen *Xanthomonas campestris* pv. *musacearum* (causing Xanthomonas wilt disease) for which there is no known genetic resistance in banana.

In this study, the transgenic plantain lines were field tested for the efficacy of the genes against nematode resistance. Eight transgenic lines provided statistically significant resistance during vegetative growth, ten lines at flowering and all twelve lines at harvest (Fig. 2). At harvest, seven lines exceeded 90% resistance to total nematodes and line P77 was 99 ± 1% resistant. At flowering, a key sampling time for assessing nematode damage^45^, the line P77 provided >90% resistance with an additional three lines offering >80% resistance, a level considered sufficient to prevent agronomic damage. Only line P77 exceeded the 80% threshold during vegetative growth with a resistance level of 90 ± 7%. The increasing resistance with age of the mother plants may relate to the stress of both the defence and the reduced suitability of older roots for the dominant nematode *R. similis* causing a decline in its rate of reproduction.

All of the transgenic lines, except C15, evaluated in the field showed expression of cystatin or/and peptide, whereas no expression of transgene was observed in control non-transgenic plants. All the dual lines expressed both the cystatin and the peptide. The expression of both transgenes varied among the different transgenic lines tested in the current study suggesting that the transgenes were integrated randomly at different transcriptionally active sites in the plantain genome, though no correlation was observed between level of gene expression and level of nematode resistance.

This study demonstrated the effect of the transgenic resistance on *H. multicinctus*, an important nematode pest of plantain, especially in West Africa^23^, but also present elsewhere across *Musa* growing regions^16^. Individuals of this genus do contribute to root necrosis of plantain cultivar ‘Gonja manjaya' but to a much lesser extent than *R. similis*^22^*. H. multicinctus* was abundant only in the roots of the control plants with added nematodes with a mean of 3,865 ± 1,492/100 g of root, which was 28 ± 11% of total nematodes at flowering time. However, their population was much lower (only 4 ± 2% and 6 ± 2%) during vegetative growth and at harvest. The low number and reduced capacity of *H. multicinctus* to cause necrosis justified using the density of just *R. similis* in the analysis shown in Fig. 3. The asymptotic curve created using the current data (Fig. 3) indicates that 317 *R. similis*/100 g roots are responsible for 10% necrosis. This is similar to a value of 273 *R. similis*/100 g roots derived previously^46^ using data collected from East African highland banana in Uganda^47^. Our study demonstrated resistance against a combined population of two key nematode species from two genera, which is very encouraging. Assessment against other species, in particular *Meloidogyne* spp. and *Pratylenchus* spp. is essential and promising lines will be further evaluated against these nematodes in a subsequent trial to confirm the true potential of this resistance.

The current study re-emphasises the considerable impact of nematodes on plantain, with the nematode-inoculated control non-transgenic plants of ‘Gonja manjaya' yielding lower relative bunch weights (about 33%) than control plants without nematode inoculation (Table 1). The bunch weights were also significantly greater in the nematode challenged transgenic line P78 compared with non-transgenic control plants. However, this comparison of harvested bunch weights underestimates the considerable effect of nematodes, as a key additional component of their damage is loss of plants and bunches after toppling. This occurs as a result of nematode root damage and consequent loss of plant anchorage. Additionally, it has recently been established that nematode infestation is directly related to snapping of plantain stems, when experiencing periods of water stress^23^, a result of poor pseudostem turgor due to lower water uptake of impaired roots^48^. The proportion of yielding plants of lines P53 and P77 was significantly greater than that for the non-transgenic controls with added nematodes (Table 1). The product of bunch weight and proportion of plants harvested indicates that five lines provided greater yield than the controls with added nematodes. They were D66, P46, P53, P77 and P78 with relative yields/planting of 104%, 127%, 169%, 186% and 144% respectively in comparison to controls with added nematodes. The corresponding value for control plants to which nematodes were not added was 136% as some plants in this group did topple. The benefit provided by some lines, particularly line P77, is considerable.

Previous work related to the current study established a cubic curve relating increasing nematode density with a reduction in leaf area index (LAI)^22^. A similar but linear effect was observed in the current study, with 10% loss in LAI being associated with 850 nematodes/100 g roots. This is a lower estimate than the previous assessment, possibly because *R. similis* was the predominant nematode on the plantains in the current study rather than *H. multicinctus* as reported previously. Environmental variables such as season and site differences are also likely to affect this relationship. The regression curve in this study suggests that the nematode suppression of LAI by 10% was associated with a 13% loss in yield. Overall the results confirm LAI is a potentially useful early indicator of subsequent yield loss due to nematode challenge.

While the value of a nematode resistant plantain focuses on the levels of nematode population reduction, other factors are also critical, such as plant vigour and yield, reduction in root necrosis, root death, and toppling due to nematodes. An overview of the lines of interest for all data on plant growth and nematode resistance was obtained using Cluster analysis. This approach has been used before to compare plants of transgenic lines^49^. Four of the lines which provided high resistance clustered together with the control plants without added nematodes (D14, D66, P77 and P78, Fig. 5). These four best lines will be further evaluated for trait stability, whereas the remaining lines hold less interest. The cystatin line C6 did rank 6^th^ overall for resistance but provided a lower bunch weight in comparison to control plants with added nematodes. In previous work, a rice cystatin provided 70 ± 10% resistance to *R. similis* in a glasshouse trial with young ‘Cavendish' dessert banana^35^. This is a similar resistance level to that of line C6 observed during the vegetative growth phase of plantain ‘Gonja manjaya'. However, there was no significant difference, in resistance of plantains when all dual lines and peptide lines were compared by Oneway ANOVA with *apriori* contrasts. Possibly, the cystatin contributed little in dual lines to the peptide-mediated resistance and so further research is required to ensure plantains express effective levels of both defences to help assure durability in the field.

In order to assess the durability of nematode resistance and yield performance, we are collecting data for the 1^st^ and 2^nd^ ratoon crops in the ongoing field trial. This trial will be expanded to multi-locations to capture the different environment effect on nematode resistance for the best lines. It is well known that pests can evolve and “breakdown” resistance of transgenic plants in the field^50^. We have plans to study comprehensively the complex issues by which nematodes may circumvent resistance, before the plants are made available to growers. We have also included transgenic lines with dual genes in this study to strengthen durability.

Successful uptake and acceptance of a transgenic crop depends upon a number of factors, but will principally depend upon the value of losses prevented and availability of transformed, locally adapted crop varieties^51^. This study demonstrates a transgenic approach that reduces considerable nematode-related yield loss for plantain cultivar ‘Gonja manjaya', which is widely grown in Africa. The benefits from nematode control on *Musa* in West Africa are probably similar to estimates for Uganda of >$250 M over 30 years with non-adoption costs of $179–365 M^52^,^53^. That potential requires a demonstration of effective resistance to all economically important nematode pests in the different environmental conditions of those African countries interested in adopting the approach. Placing the peptide under control of a root-specific promoter may be a technological improvement of value as has already been achieved for potato^54^. The scientific evidence base must be completed to meet all requirements of the national biosafety committees of those countries seeking to adopt the approach. Subsequent uptake requires investment to reduce the yield gap^51^ including effective government policies that support their adoption by small farmers^55^. Policy makers concerned about food security should consider GM approaches when other solutions to problems identified are unavailable^56^. Here we offer an effective, low cost approach to the previously intractable problem of nematode control that could reduce the yield gap to small farmers without altering their cropping choices. We present strong evidence for the uptake of this royalty-free technology in Africa to support the future food security of plantain-dependent Africans.

## Methods

### Plant Materials

Two hundred and forty-five independent transgenic lines of plantain cultivar ‘Gonja manjaya' (*Musa* spp., AAB group) genetically modified to express maize cystatin that limits nematode digestion of dietary protein or synthetic peptide that disrupts nematode chemoreception or both these traits stacked together were generated and analysed in previous work^36^. Twelve transgenic lines (five lines with synthetic peptide, two lines with maize cystatin and five lines with dual stacked genes) showing presence and expression of transgene and high resistance against nematodes in screen house pot trials were advanced to a confined field trial in Uganda after approval from NBC (Decision Number 1/2012).

### Plant preparation for field trial

Transgenic and non-transgenic control plantlets were micropropagated on proliferation medium [MS salts and vitamins^57^, 10 mg/l ascorbic acid, 100 mg/l myo-inositol, 5 mg/l BAP, 30 g/l sucrose, 3 g/l gelrite, pH 5.8] in order to generate 20 clones of each line. The individual shoots were transferred to rooting medium (MS salts and vitamins, 10 mg/l ascorbic acid, 100 mg/l myo-inositol, 1 mg/l IBA, 30 g/l sucrose, 3 g/l gelrite, pH 5.8). The well-rooted transgenic and control non-transgenic plants were weaned in small disposable plastic cups (10 cm diameter) containing sterile soil, transferred to a transparent polythene chamber within a contained (Biosafety level II) glasshouse and grew for 4 weeks under diffused light, high humidity and 26–28°C. After 3 weeks, humidity was progressively reduced by gradual opening of the chamber's side. After 4 weeks, plants were transferred to 30 cm diameter pots in the glasshouse and irrigated manually on alternate days. Two-month old plants were moved to a screen house and infected with a mixed population of nematodes.

### Infection of potted plants with nematodes

Roots were collected from bananas growing at a nematode-infested banana site at Sendusu, National Agricultural Crops Research Institute, Namulonge in Central Uganda, chopped into small size pieces and mixed thoroughly before using sub-samples for plant infection. Nematode densities were estimated in several 5 g root samples as before^36^. The nematode population determined from 5 g of roots was a mixture of 77.3% *R. similis*, 17.2% *H. multicinctus* and 5.5% motile stages of *Meloidogyne* spp. Each plant to be infected received 1,000 nematodes in 3.5 g of chopped roots, which was incorporated in the potting soil. The plants were then grown in the screen house for 1 month before transplanting to the field.

### Field preparation, trial design, planting, maintenance and harvesting

A replicated trial of 12 transgenic lines of plantain cultivar ‘Gonja manjaya', along with control non-transgenic plants was planted in December 2012. The trial was in a confined field at the National Agriculture Research Laboratories (NARL), Kawanda, Uganda.

The field was prepared by ploughing twice before three month old plants were transplanted into holes of 30 cm diameter and depth and spaced 3 m × 3 m apart. The trial was a randomized complete block design with four replicates of each transgenic line in each of 4 blocks (a total of 16 plants of each line) plus two sets of replicates/block (a total of 32 plants) for the control plants with added nematodes. Twenty replicates of non-transgenic controls that had no nematodes added before planting were placed in separate block adjacent to blocks of treatment plots rather than within them in order to prevent inward migration of the added nematodes. Tissue culture plants of the same size were planted to form a border row of guard plants around the trial. Plants were watered daily for 1 month after transplanting and three times per week thereafter until well established before relying only on rainfall. The trial was maintained using recommended farming practices (weeding, de-suckering, mulching and adding manure). Hand weeding and de-suckering was conducted on a monthly basis. No chemical fungicide or nematicide was applied. All the plants in the trial, including the non-transgenic control plants and guard rows were inspected for flowering daily after 9 months of planting. Upon emergence, each inflorescence was bagged and male flowers were removed when they formed. The plants were observed by visual inspection for structural abnormalities and data were collected on agronomic performance, nematode counts and root necrosis.

### Nematode Count and Damage Assessment

Samples for nematode count and root necrosis of individual plants were collected at 4, 7, 10 MAP and at both flowering and harvesting stages. Root samples were collected from each plant, cleaned of soil and weighed. Nematodes were extracted from two 50 g sub-samples of randomly selected pieces of each root system. Each sub-sample was macerated in a blender for 10 s and the nematodes extracted^36^. Nematode population densities were estimated from three replicates of 2 ml aliquots taken from a 25 ml suspension of each sample. Nematodes were identified to species and population densities estimated per 100 g root fresh weight. Root damage was assessed by calculating the percentage dead primary roots (completely rotten or shrivelled) and root necrosis of five randomly selected functional roots (showing at least some healthy tissue) from each plant using a standard approach^36^.

### Data collection for agronomic and yield performance

Plant growth was assessed every month starting at 5 MAP. Data were collected for each plant on plant height, pseudostem girth, number of functional leaves, timings of flowering and harvest. At harvest, data on bunch weight, number of hands, number of fingers and weight of individual fruit were recorded. Total leaf area (TLA) was estimated using the equation TLA = n (0.411G + 0.381H − 0.404) where n is the number of functional leaves more than 50% green and fully attached to the pseudostem, G is girth (cm) at the base of the pseudostem and H is the plant height (cm) measured from the base to the axil of the topmost pair of fully expanded leaves^58^. LAI was also assessed for all plants in replicates of four plants per image by digital hemispherical photography when the plants were at 9–12 MAP, as previously described^22^.

### Molecular Characterization

#### RNA extraction, RT and qRT-PCR analysis

Total RNA was extracted from 100 mg root tissue of transgenic plants of 10 lines (D12, D14, D30, D46, D66, P46, P48, P53, P77 and P78) and control non-transgenic plant with nematode infestation from the confined field trial using the RNeasy plant mini kit (Qiagen, GmbH, Hilden, Germany) and treated with DNase. The quantity and quality (A260/230 and A260/280) of extracted total RNA were determined using the Nanodrop 2000. RNA was checked with PCR for absence of genomic DNA. Complementary DNA (cDNA) was synthesized from 1 μg of total RNA using reverse transcriptase of the Maxima H Minus First Strand cDNA synthesis kit with oligo dT primers (Thermo Scientific). Reverse transcriptase PCR (RT-PCR) was performed with 1 μl of each cDNA synthesized using primers specific to the peptide gene (forward primer 5′-TACTCAACGAAGGGCAAACC - 3′ and reverse primer 5′- GCATAGTAGTACAAGCGGAGAC-3′). Amplification of the banana *25S* ribosomal transcript, used as an internal control to determine the quality of RNA, was performed using the forward primer (forward primer: 5′ -ACATTGTCAGGTGGGGAGTT- 3′ and reverse primer: 5′ -CCTTTTGTTCCACACGAGATT- 3′).

Real-time RT-PCR was carried out using 7900 Real Time PCR System (Applied Biosystems, USA) using Maxima SYBR green/ROX PCR kit (Thermo Scientific) according to the manufacturer's instructions. qRT-PCR was performed with 1 μl of each cDNA synthesized at 1:10 dilution using synthetic peptide specific primers as described above. Two independent biologically replicated experiments were set up with three technical replicates in each experiment. No-template controls and a non-transgenic control were included. Relative expression data were normalized using the banana *25S* ribosomal gene specific primers and non-transgenic control plants acted as calibrator to calculate relative expression level of peptide in transgenic plants. The levels of peptide were obtained using the 2^−ΔΔCt^ method^59^ relative to the transgenic line providing the lowest expression level.

#### Protein extraction and western blotting

Young root tips were collected from the field for plants of the seven transgenic lines (C6, C15, D12, D14, D30, D46 and D66) that harboured the cystatin gene. Samples from the roots of plants in each block were pooled. Western blots were carried out as described previously^36^.

### Statistical Analysis

All data were analysed using a standard statistical package (SPSS v20; IBM Corporation Armonk, New York, USA; http://www-01.ibm.com/software/analytics/spss). The choice of analysis used for data was informed by both the help files of the package and a standard text^60^. Nematode densities were transformed to square root values and proportions to arcsin values before analysis and back-transformed for presentation. All means are given with the standard error of the mean (SEM). The analyses carried out were: χ^2^, ANOVA using the general linear model univariate procedure and One-way analysis with both *apriori* contrasts and *post-hoc* comparisons of means. Both linear and non-linear regression and hierarchical cluster analysis were also applied. Cluster analysis was carried out using Ward's method and the measure of squared Euclidean distance. Values were transformed to Z values and transformation method involved rescaling 0–1.

## Author Contributions

L.T. and H.J.A. designed the experiments, provided supervision and co-wrote the manuscript. L.T. and J.N.T. developed cell suspension and generated transgenic plants. L.T. and A.B. established and conducted the field trial. A.B. collected the data and H.J.A. analysed it. H.R. made the constructs under the guidance of P.E.U. and carried out molecular characterisation with A.B. and J.N.T. C.C. and W.K.T. managed the trial and D.C. supplied nematodes and advised on nematological aspects of the work. All authors discussed the results and commented on the manuscript.

## Figures and Tables

**Table 1:** Agronomic performance of the mother crop of transgenic plantain lines grown in the confined field trial

Line	Days to flowering	Height (cm)	Girth (cm)	No. of functional leaves	Leaf area (m^2^)	Bunch weight (kg)	No. of hands	No. of fingers	Weight of single fruit (g)	Yielding plants	Root necrosis† (%)
+nem	340.2 ± 7.1	242.0 ± 8.5	58.4 ± 1.4	7.7 ± 0.4	7.4 ± 0.5	9.8 ± 0.8	7.5 ± 0.2	74.5 ± 2.8	164.5 ± 9.3	21/32	21.4 ± 0.01
C15	347.5 ± 12.0	229.3 ± 10.7	57.7 ± 1.9	8.5 ± 0.5	7.8 ± 0.7	2.5 ± 0.5***	6.6 ± 0.3**	64.6 ± 4.4	140.3 ± 7.2*	12/16	27.4 ± 0.02
C6	348.6 ± 8.7	217.8 ± 15.9	54.5 ± 2.4	9.4 ± 1.9	7.4 ± 0.8	6.5 ± 0.7**	7.1 ± 0.2	72.0 ± 3.2	133.9 ± 7.9**	12/16	20.9 ± 0.02
D12	396.5 ± 10.6***	206.0 ± 19.5*	52.3 ± 2.7	7.2 ± 0.7	6.0 ± 1.1	7.1 ± 0.9*	4.8 ± 0.3***	35.8 ± 3.7***	95.0 ± 10.1***	4/16††	36.4 ± 0.02***
D14	338.8 ± 9.5	247.6 ± 13.0	59.2 ± 1.1	10.1 ± 0.4***	9.8 ± 0.6*	7.6 ± 1.9	7.3 ± 0.2	76.1 ± 4.6	166.1 ± 10.1	10/16	21.4 ± 0.02
D30	353.2 ± 15.4*	210.7 ± 16.8	57.2 ± 1.7	7.8 ± 0.5	6.7 ± 1.0	7.7 ± 1.0	7.0 ± 0.3	69.8 ± 7.5	121.9 ± 23.8**	5/16†	27.4 ± 0.02
D46	364.9 ± 11.1	234.2 ± 13.1	58.8 ± 2.5	8.2 ± 0.5	7.6 ± 0.7	7.9 ± 1.0	7.3 ± 0.2	71.5 ± 2.9	137.3 ± 9.0*	12/16	21.3 ± 0.02
D66	346.5 ± 7.4	255.8 ± 9.8	62.5 ± 2.6	10.2 ± 2.1	9.9 ± 1.7*	8.2 ± 1.2	7.4 ± 0.1	72.7 ± 2.6	142.1 ± 4.7*	13/16	18.9 ± 0.02
P46	352.4 ± 7.6	212.8 ± 7.3*	57.1 ± 1.3	7.6 ± 0.4	6.4 ± 0.4	9.3 ± 0.8	7.1 ± 0.2	78.3 ± 8.2	140.9 ± 8.5*	14/16	18.8 ± 0.02
P48	340.0 ± 16.8	240.7 ± 14.1	55.4 ± 4.3	11.7 ± 2.9	10.0 ± 1.5*	9.3 ± 0.9	7.0 ± 0.4	68.1 ± 4.0	136.6 ± 7.1*	8/16	26.3 ± 0.02
P53	340.6 ± 7.6	221.8 ± 8.8	57.3 ± 1.5	7.4 ± 0.4	6.5 ± 0.6	11.5 ± 1.0	6.9 ± 0.2	69.2 ± 2.4	153.3 ± 8.0	15/16†	16.4 ± 0.02
P77	336.4 ± 8.6	265.2 ± 7.5	60.0 ± 1.2	8.3 ± 0.6	8.7 ± 0.7	11.9 ± 1.3	8.1 ± 0.2*	83.4 ± 4.2	174.8 ± 9.0	16/16††	07.6 ± 0.02***
P78	344.2 ± 7.0	247.7 ± 9.4	58.7 ± 1.9	9.5 ± 0.4**	9.2 ± 0.5	12.3 ± 1.1*	7.8 ± 0.3	77.3 ± 1.7	161.1 ± 6.0	12/16	16.5 ± 0.02
−nem	305.3 ± 4.7**	280.6 ± 5.1**	61.8 ± 0.9	9.7 ± 0.2***	10.7 ± 0.3***	14.6 ± 0.5***	7.8 ± 0.2	88.8 ± 2.9**	156.8 ± 5.2	12/20	02.3 ± 0.02

Values for agronomic parameters are means ± SEM for all variables but the number of yielding plants. One-way analysis of variance with *a priori* contrasts was used to compare means with the corresponding value for the control to which nematodes were added prior to planting (+nem). Means that indicate a significantly different performance than the +nem control are indicated by asterisk (*, P < 0.05; **, P < 0.01; ***, P < 0.001). The column for yielding plants shows the number yielding plants/total planted. χ^2^ analysis was used to identify those transgenic lines that provided significantly more or less yielding plants than expected relative to the +nem control (^††^, P < 0.01; ^†^, P < 0.05). −nem indicates control non-transgenic plants to which nematodes were not added before planting.

^†^Data and analysis for root necrosis is as for Fig. 3.
